# New Evidence for the Contemporary Presence of Juvenile White Sharks (*Carcharodon carcharias*) in the Adriatic Sea

**DOI:** 10.3390/fishes10010025

**Published:** 2025-01-08

**Authors:** Patrick L. Jambura, Pero Ugarković, Mišo Pavičić, Ilija Ćetković, Simone Niedermüller, Jürgen Kriwet, Julia Türtscher

**Affiliations:** 1 Department of Palaeontology, University of Vienna, 1090 Vienna, Austria; 2 The MECO Project, 546 45 Thessaloniki, Greece; 3 Independent Researcher, 21000 Split, Croatia; 4 Institute of Oceanography and Fisheries, 21000 Split, Croatia; 5 Institute of Marine Biology, University of Montenegro, 85330 Kotor, Montenegro; 6 World Wide Fund for Nature Mediterranean Marine Initiative (WWF MMI), 00161 Rome, Italy; 7 Vienna Doctoral School of Ecology & Evolution (VDSEE), University of Vienna, 1030 Vienna, Austria

**Keywords:** Chondrichthyes, Mediterranean Sea, nursery area, citizen science, social media, threatened species

## Abstract

The presence of the white shark (*Carcharodon carcharias)* in the Mediterranean Sea is well documented, but mainly through historical and opportunistic records. Historically, the Adriatic Sea, particularly its eastern coastline, was considered a hotspot for white sharks, with relatively frequent reports of juvenile specimens suggesting a potential nursery area. However, since the second half of the 20th century, the abundance of white sharks in the Adriatic has experienced a dramatic decline, with the last confirmed sighting recorded in 2011. In this study, we report the recent capture of a young-of-the-year (YOY) white shark off the Croatian coast, previously misidentified as a porbeagle shark (*Lamna nasus*). In addition, we revisit historical records of white sharks in the Adriatic Sea to assess whether this region can be considered a nursery area. Our findings highlight significant gaps in the understanding of white shark spatial ecology and reproductive biology in the Mediterranean Sea. Furthermore, this study emphasizes the critical role citizen science and public engagement can play in documenting occurrences of these elusive and endangered predators, offering valuable insights for future conservation efforts.

## 1 Introduction

The white shark, *Carcharodon carcharias* (L., 1758), is a large, cosmopolitan apex predator that can reach a total length of up to 6 m [1,2]. This species is known to occupy a wide range of different habitats, from shallow coastal waters to continental shelves and remote islands, with long periods spent in pelagic habitats during mid-ocean migrations [3–5]. Despite being highly migratory and known for their offshore and transoceanic migration patterns [6,7], there are several populations within three main lineages of white sharks that exhibit little to no genetic exchange ([8] and references therein).

The Mediterranean population of white sharks is probably the most threatened among these populations and is classified as critically endangered (CR) by the IUCN Red List of Threatened Species [9]. Mediterranean white sharks show a complex trajectory of population change, marked by an initial historical increase, followed by a sharp decline of 61% since the second half of the 20th century alongside regional declines of 52–96% and a significant contraction in spatial distribution [10]. Although attempts have been made to study the Mediterranean population in the field [11–13], these have remained unsuccessful, likely due to the low population density and lack of (known) aggregation sites. Consequently, most of our knowledge of the biology and ecology as well as the population status of Mediterranean white sharks relies exclusively on opportunistic records and meta-analyses that compiled these records [10,14–18].

The Adriatic Sea, an elongated basin located in the Central Mediterranean Sea between the Italian peninsula and the Balkans, has historically been regarded as a hotspot for white sharks [18], particularly along the Eastern Adriatic coast [19,20]. Due to the relatively high occurrence of juvenile white sharks, the region was also considered a potential historical nursery area for the species [18]. However, over the past three generations (~69 years), the abundance of white sharks in the Adriatic Sea has declined dramatically by an estimated 84% [15]. Today, sightings are rare, with the last confirmed record occurring in 2011 when a juvenile white shark of approximately 220 cm total length (TL) was caught off the coast of Bar, Montenegro [18].

In this study, we present data on a recent capture of a young-of-the-year (YOY) white shark off the coast of Croatia, which was previously misidentified as a porbeagle shark, *Lamna nasus* [20]. Photographic evidence is provided to clearly demonstrate key morphological features that confirm our species identification. Furthermore, we review previous records of white sharks in the Adriatic Sea and discuss whether this region can indeed be considered a historical or current nursery area for white sharks.

## 2 Materials and Methods

The record reported here was collected through the citizen science initiative “The MECO Project” [21]. This initiative uses a verified citizen science model, where citizen-submitted observations are verified by scientists with taxonomic expertise (see Gardiner et al. [22]). Subsequently, interviews are conducted to confirm the reported data and to obtain further information. The MECO project datasheet includes the following data categories: date, time, location, report type (e.g., fishing, scuba diving, snorkeling, etc.), media (e.g., social media, direct message, newspaper), sex, ontogenetic stage, condition (dead or alive), total length (TL), depth, temperature, coordinates, reporter, and remarks.

In addition, an extensive literature search was conducted to compile an updated list of records of white sharks in the Adriatic Sea using Google Scholar and the Zoological Record (Appendix A; Table S1). Several published and recited sightings, which are based on oral communications alone and are not accompanied by further evidence, are not included in Appendix A as they could not be validated (see Supplementary Materials Table S2). All new records had to be accompanied by photographic evidence to confirm species identification. Species identification was based on the following features: (1) a heavy, long-snouted, spindle-shaped body; (2) a blunt, conical snout; (3) strong keels on the caudal peduncle; (4) the absence of a secondary caudal keel; (5) a large first dorsal fin, very small second dorsal and anal fins; (6) a lunate caudal fin; (7) large, flat, triangular, serrated teeth; (8) long gill slits; (9) small black eyes; (10) a sharp color change from greyish dorsally to white ventrally; and (11) pectoral fins with black tips on the ventral side [5,23,24]. When applicable, the ontogenetic stages were identified based on the total length following Boldrocchi et al. [18]: young-of-the-year (YOY; TL ≤ 1.75 m), juvenile (TL 1.75–3.0 m), subadult (♂TL 3.0–3.6 m; ♀TL 3.0–4.5 m), and adult (♂TL > 3.6 m; ♀TL > 4.5 m).

## 3 Results and Discussion

On 12 September 2023, a white shark was caught approximately 4 NM southwest of the island of Svilan near Rogoznica (N 43°29′29.037204 E 15°44′25.207208). It was caught between 16:00 and 19:00 with a bottom-set long line at a depth of 100–120 m. The specimen was approximately 1.20–1.30 m in total length and weighed ca. 20 kg, identifying it as a YOY white shark. This is also consistent with the shape of the first dorsal fin, which had a rounded apex, a feature typically observed in embryonic and neonate white sharks up to 1.6–1.7 m [25].

**Figure 1:**
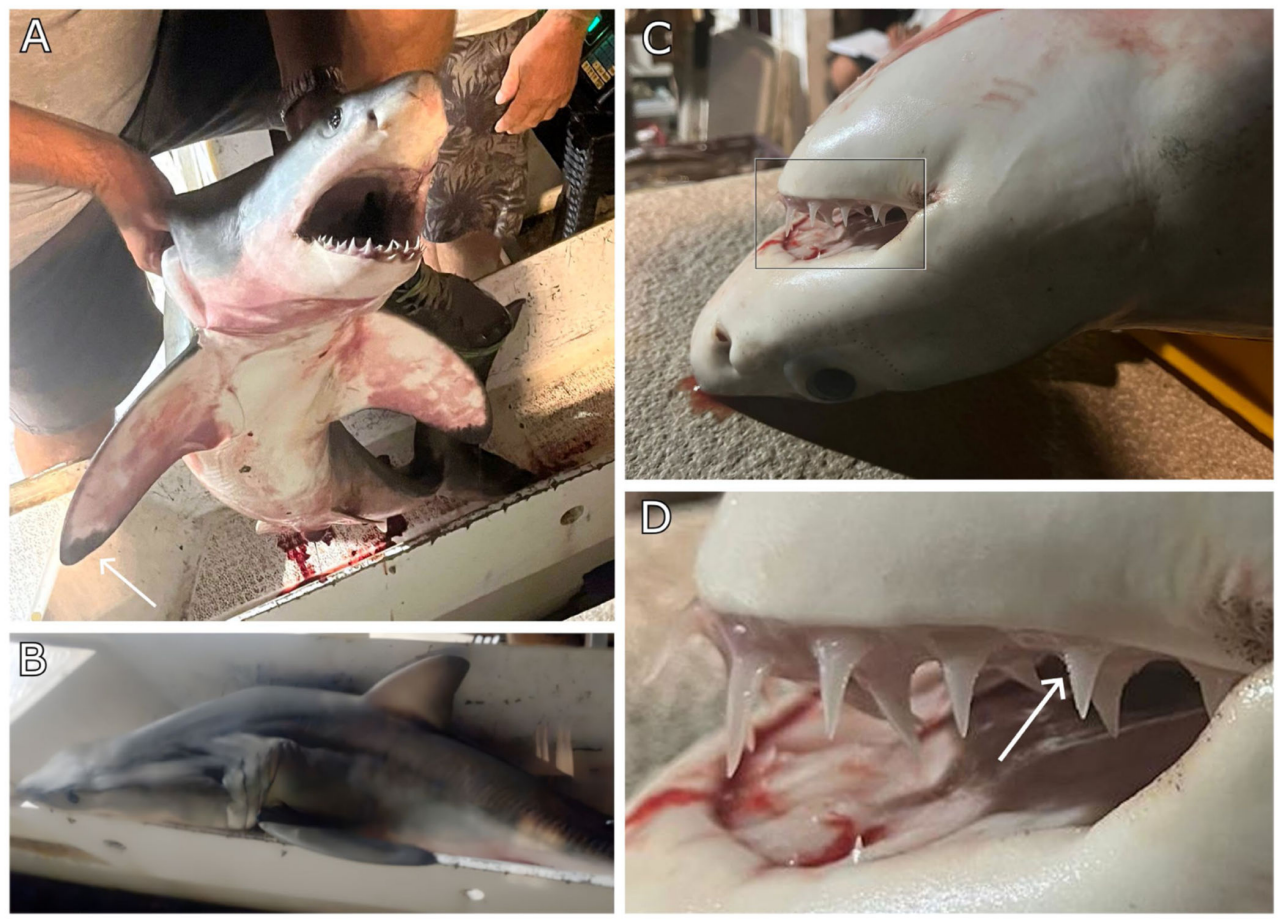
The young-of-the-year (YOY) white shark (*Carcharodon carcharias*) caught off Rogoznica, Croatia, on 12. September 2023. (**A**) The ventral side, showcasing the black tips of the pectoral fins; (**B**) a lateral view showing the rounded apex of the first dorsal fin, which lacks white markings at the rear edge; and (**C**) a close-up of the head and (**D**) jaws, showing triangular teeth with serrations and no cusplets. Figure 1 was reproduced from https://www.morski.hr/kod-rogoznice-ulovljena-velika-bijela-psina-ribari-je-prodali-iako-je-zasticena-vrsta/#google_vignette, with permission from the publisher, 2024 (accessed on 2 August 2024).

A recent review of white shark occurrence in the eastern Adriatic Sea [20] mentioned this record, but identified it as a porbeagle shark, *Lamna nasus*. An interview with the fisherman who caught the shark clarified that the date and location reported in newspapers and in Soldo and Bakiu [20] were slightly inaccurate, with the incident actually occurring two days earlier than previously stated. The presence of a conical snout, serrated teeth, and the lack of lateral cusplets, along with distinctive black markings on the ventral side of the pectoral fin and the absence of a secondary caudal keel as well as the lack of a distinctive white free rear tip on the first dorsal fin (Figure 1) unambiguously demonstrate that this shark was indeed a YOY white shark and not a porbeagle shark, which is also known to occur in this area [26–28].

Soldo and Bakiu [20] further discuss that this would have been the first record of a juvenile white shark in the Adriatic Sea, which is inconsistent with previously reported records included in their list. In fact, 13 YOY and juvenile white sharks have been reported in the Adriatic Sea since 1868, accounting for approximately 15.5% of all records of white sharks with a known ontogenetic stage (seven YOY, six juveniles, six subadults, 14 subadult/adults, 49 adults, and 14 of an unknown ontogenetic stage; Appendix A). However, the majority of these records are based on historical accounts for which there is no physical or photographic evidence. Only three other records of older juvenile white sharks in the Adriatic Sea (nos. 54, 88, 89; Table S1) are supported by photographic documentation. Therefore, the specimen reported here represents the only unambiguous record of a YOY in the Adriatic Sea.

The white shark caught in Rogoznica is the first record of a white shark in the Adriatic Sea since 2011. The presence of white sharks in the Adriatic Sea, especially along the eastern part of the basin, has previously been associated with the historically high abundance of Atlantic bluefin tuna *Thunnus thynnus* in this area, which are suggested to be the primary prey for white sharks in the Mediterranean Sea [19]. White sharks are generalist top predators that feed on teleosts [29,30], cephalopods [29,31], elasmobranchs [29,32], and mammals [29,33,34]. However, their nutritional niche breadth increases during ontogeny, with only adult white sharks preying on higher-trophic species, while juveniles primarily feed on lower-trophic teleosts and elasmobranchs [29,30,32]. Therefore, the availability of tuna might explain the presence of adult white sharks, which have been frequently observed in close proximity to tuna fisheries [35–39], but does not account for the occurrence of juvenile white sharks in these waters.

**Figure 2:**
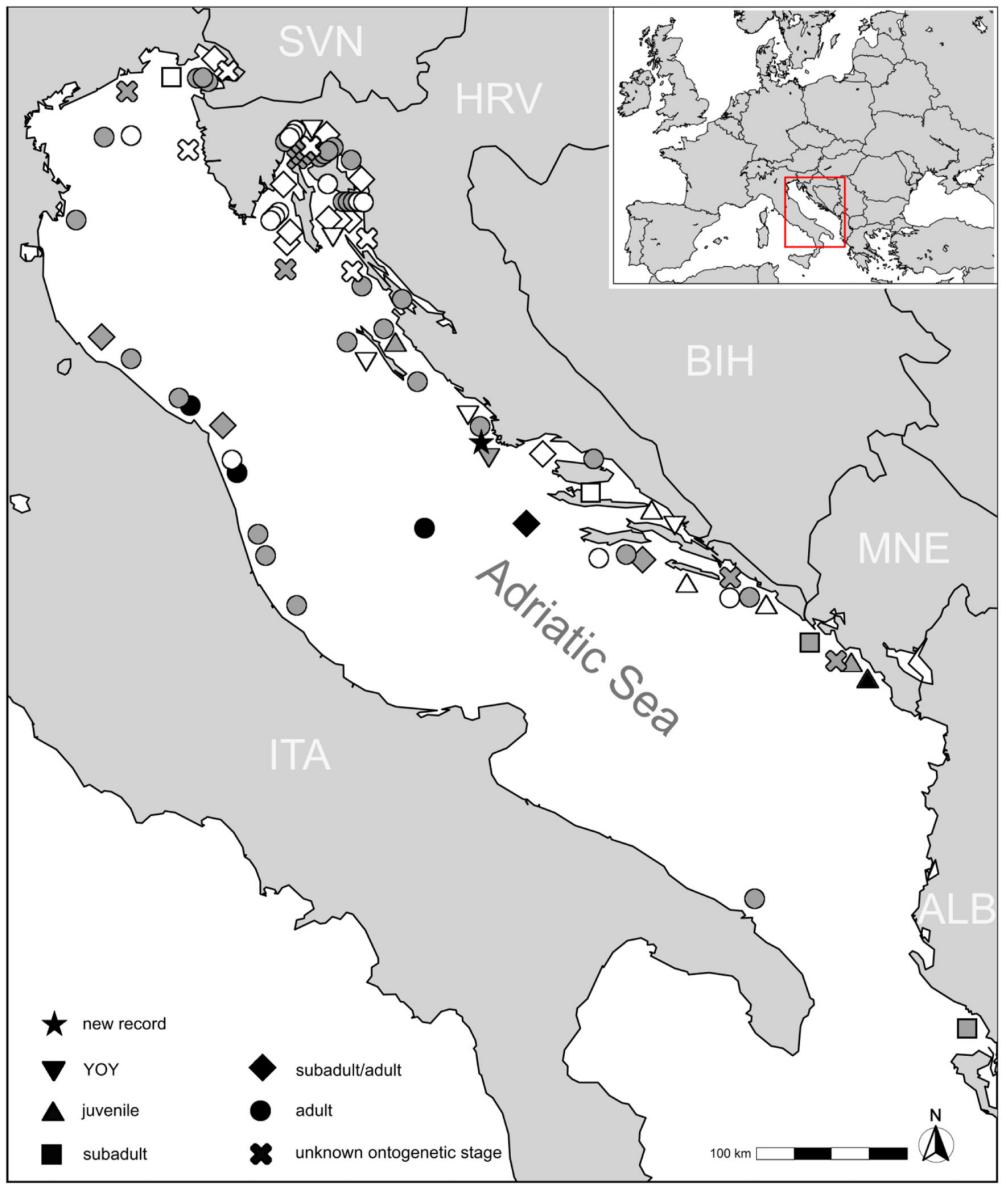
The spatial distribution of white shark (*Carcharodon carcharias*) sightings in the Adriatic Sea. Colors indicate the relative age of each record: white, 19th century; grey, 20th century; and black, 21st century. Country abbreviations follow ISO 3166-1 standards.

An examination of the collected data reveals that juveniles exhibit a distinct distribution pattern compared to subadult and adult individuals. While adult (and possibly large subadult) white sharks have been recorded throughout the Adriatic Sea, juveniles appear to be restricted to the eastern coast, suggesting specific ecological or environmental factors influencing their distribution (Figure 2; Appendix A). The presence of numerous offshore islands in close proximity to the coast along the eastern Adriatic Sea likely provides a suitable habitat for juvenile white sharks, as has been suggested for other Mediterranean regions [40]. Notably, there is no specific hotspot along the eastern coast where juveniles are more frequently recorded. Instead, sightings are distributed along the entire coastline. This observed pattern cannot be attributed to differences in sampling effort between the eastern and western coasts, as Italy, located on the western Adriatic coast, operates the largest fishing fleet in the region [41].

Previous reports from the western Adriatic coast have suggested the presence of juvenile white sharks also in this area [16,42]. Most of these records date back to the period 1872–1905 when the Imperial Maritime Austrian government issued three circulars offering monetary rewards for captured white sharks [16]. In order to claim the reward, captured specimens had to be presented to the Natural History Museum in Trieste for identification. Unfortunately, white sharks were not the only species that were mentioned in these circulars, and payment records preserved in the State Archive of Trieste do not specify the species for which they were issued [16]. Therefore, the identification of these specimens is uncertain, and we were unable to verify these records (Table S2). A more recent record from Termoli (Italy) mentions the capture of 4–5 female juvenile white sharks, but this record is based on informal communication and lacks supporting evidence [14,16,18]. Consequently, all confirmed records of juvenile white sharks in the Adriatic Sea come from the eastern coast.

The Adriatic Sea has previously been suggested as a potential nursery area for white sharks due to the relatively high occurrence of juvenile specimens in this region [18]. Based on its size and caudal fin morphology, the specimen reported here was identified as a YOY, suggesting that parturition may occur in the Adriatic Sea. This is further supported by a historical record of a gravid female caught near Rijeka [20]. Similarly, several other areas in the Mediterranean Sea have been proposed as potential nursery areas for white sharks, i.e., the Sicilian Channel, Italy [14]; the Gulf of Gabes, Tunisia [14,43,44]; and Edremit Bay, Turkey [45], based on increased juvenile occurrences. However, no discrete nursery areas have been definitively identified in the Mediterranean Sea yet [25]. Furthermore, while white sharks are known to exhibit philopatry [46–48], there is evidence that pregnant females are not restricted to a single nursery area but may use widely separated pupping areas [49], and that parturition likely occurs over broader horizontal and vertical spatial scales [7,25]. This was also suggested by previous reports of YOY and juvenile white sharks along the Libyan coast, which were outside their presumed nursery area in the Gulf of Gabes, indicating a wider distribution of these early ontogenetic stages than was previously thought [50]. This indicates that the Mediterranean white shark population exhibits a more complex spatial ecology than previously thought. While the Adriatic Sea remains a plausible candidate for a nursery area, more data on white shark occurrences and broader studies on their spatial ecology in the Mediterranean Sea are needed to confirm this hypothesis.

The record of this YOY white shark in the Adriatic Sea highlights the knowledge gaps that still exist regarding the distribution, spatial ecology, and reproductive biology of Mediterranean white sharks. Citizen science can help to address this data deficiency by providing opportunities to collect new observations that would otherwise go undocumented and should be considered as a complementary approach to studying the biology of these elusive animals in the Mediterranean Sea.

In addition, citizen science also has great potential to promote conservation by raising public awareness of the presence of this species in the Adriatic Sea. The white shark is protected under several international agreements, including the Convention of Migratory Species (Appendix I and II) and the Bern Convention. In addition, white sharks are protected via regional binding decisions (e.g., UNEP MAP SPA/BD Protocol, Decision IG.26/4) and are listed as a prohibited species for EU member states in all waters (Regulation (EU) 2019/1241). Mediterranean countries are required to provide high protection against fishing, ensuring white sharks are released unharmed whenever possible. The retention, transshipment, landing, sale, or display of white sharks is strictly prohibited (GFCM 2018, 2021). Unfortunately, despite these regulations, white sharks and other protected species often go unreported and are illegally sold [51], as was the case with the juvenile white shark from Rogoznica, which was sold to a local restaurant. By leveraging social media and citizen science, we are not only able to collect crucial data about the Mediterranean white shark population, but also educate fishermen and the public, as well as decision makers and control authorities, about the ecological importance of this species, thereby raising awareness and encouraging conservation efforts for this iconic but critically endangered predator in the Mediterranean Sea.

## Supplementary Material

Supplementary Material The following supporting information can be downloaded at www.mdpi.com/xxx/s1, Table S1: Verified records of white sharks (Carcharodon carcharias) from the Adriatic Sea; Table S2: Published but unverified records of white sharks (Carcharodon carcharias) from the Adriatic Sea. References [14,16,18–20,26,35,37,42,52–70] are cited in the supplementary material.

Appendix

## Data Availability

All data used by the authors for the analysis are available in the Supplementary Materials.
