# Supplementary material for: New Evidence for the Contemporary Presence of Juvenile White Sharks (Carcharodon carcharias) in the Adriatic Sea
Source: Fishes. Author manuscript; Available in PMC 2025 Feb 28. (PMC7617451; doi:10.3390/fishes10010025)
Supplement: Appendix [file EMS203519-supplement-Appendix.pdf]

Appendix A

Verified records of white sharks (*Carcharodon carcharias*) from the Adriatic Sea, compiled from the literature and the MECO project. Detailed information and sources for each record are provided in Table S1. Unverified or doubtful records that were previously published are listed separately in Table S2.

Country	Location	Date	Size [cm]	Weight [kg]	Sex	Ontogenetic Stage
Albania	Qeparo-Borsh, Himare	15 April 1964	445	>550	F	subadult
Croatia	Jablanac	14 September 1868	-	-	-	-
	Sv. Juraj	16 December 1868	460	-	-	adult
	Rijeka	16 April 1872	490	-	-	adult
	Opuzen	12 May 1872	95	-	-	YOY
	Mljet	12 May 1872	237	-	-	juvenile
	Rijeka	8 June 1872	131	-	-	YOY
	Dugi Otok	16 June 1872	146	-	-	YOY
	Cavtat	25 July 1872	260	-	-	juvenile
	Rab	8 August 1872	130	-	-	YOY
	Ustrine-Cres	5 May 1877	460	-	-	adult
	Sveta Martin-Cres	8 May 1877	413	-	-	subadult/adult
	Osor-Cres	17 June 1878	371	-	-	subadult/adult
	Poreč	9 August 1878	-	-	-	-
	Osor	21 May 1879	382	-	-	subadult/adult
	Split	23 July 1879	402–445	-	-	subadult/adult
	Ustrine-Cres	21 October 1879	530	-	-	adult
	Gradac	5 October 1879	250	-	-	juvenile
	Rab	22 April 1881	380	-	-	subadult/adult
	Rab	16 October 1881	405	-	-	subadult/adult
	Sveta Martin-Cres	13 April 1882	529	-	-	adult
	Vrboska-Krk	13 June 1883	300	-	-	subadult
	Rab	26 September 1883	396	-	-	subadult/adult
	Korčula	3 March 1886	560	-	-	adult
	Krk	2 September 1887	470	-	-	adult
	Sv. Juraj	July 1888	470	-	-	adult
	Sušak	23 October 1888	500	3500	female	adult
	Senj	26 August 1890	440	-	-	subadult/adult

	Bakarac	15 September 1890	384	-	-	subadult/adult
	Pag	26 April 1891	-	-	-	-
	Bakarac	September 1892	450	-	-	-
	Zlarin	19 February 1893	165	-	male	YOY
	Bakar	29 August 1894	470	-	female	adult
	Dubrovnik	15 July 1901	520	-	-	adult
	Senj	21 May 1903	600	1200		adult
	Povile	30 September 1903	450	-	-	adult
	Bakarac	29 June 1906	522	-	female	adult
	Kraljevica	October 1909	550	-	-	adult
	Dugi Otok-Kornati	2 February 1920	525	1300	-	adult
	Ugljan	March 1926	500	700	-	adult
	Lumbarda	August 1926	400	500	-	subadult/adult
	Kraljevica	Summer 1926	600	1000	-	adult
	Lumbarda	October 1926	600	1800	-	adult
	Rogoznica	1931	150	-	female	YOY
	Susak Island	21 August 1934	-	-	-	-
	Kraljevica	2 September 1934	775	1100	-	adult
	Mošćenička Draga	7 September 1934	600	1000	-	adult
	Lukovo	20 July 1935	600	2500	-	adult
	Bakarac	Summer 1946	-	-	-	-
	Primošten	August 1950	700–800	-	-	adult
	Pag	2 October 1954	550	1500	-	adult
	Senj-Sveti Juraj	10 December 1955	460	-	female	adult
	Opatija	24 September 1961	-	-	-	-
	Rava	1968	200	-	-	juvenile
	Opatija	1971	-	-	-	-
	Kornati	17 August 1972	600	-	-	adult
	Luka Šipanska	26 July 1973	-	-	-	-
	Lokva Rogoznica	10 August 1974	500	-	-	adult
	Vrsi near Nin	17 June 1976	450	-	male	adult
	Island of Jabuka	24 March 2003	570	2000–2500	female	adult
	Vis	6 October 2008	~450	-	-	subadult/adult
	Rogoznica	12 September 2023	120–130	20	-	YOY
Italy	Adriatic	16 September 1823	490	-	F	adult
	Adriatic	1827 Spring	-	-	-	-
	Civitanova Marche	February 1839	580–600	1800	F	adult
	Trieste	1 September 1868	-	-	-	-
	Grado	19 April 1872	300	-	-	subadult
	Gulf of Venezia	Before 1881	490			adult
	Santa Croce di Trieste	14 September 1885	400	-	-	subadult/adult
	Trieste	1902	375	-	male	adult
	Trieste	June 1908	-	1400	-	adult
	Pescara	ca. 1945	600	-	-	adult
	Riccione	7 July 1961	450	-		subadult/adult
	Golfo di Venezia	7 June 1978	500	-	-	adult
	Caorle	June 1978	-	-	-	-
	Numana	May 1988	450	-	-	subadult/adult
	Porto Barricata	9 October 1988	550	-	-	adult
	Pesaro	September 1989	500+	-	-	adult
	Brindisi	15 September 1996	400–500	-	-	adult
	Senigallia	27 August 1998	500–600	1200	-	adult
	Giulianova	26 September 1999	600	-	-	adult

	Giulianova	September–October 1999	700	-	-	adult
	Falconara	30 July 2001	550–600	-	-	adult
	Porto San Giorgio	9 September 2002	700–800			adult
Montenegro	Herceg Novi	June 1926	300	-	-	subadult
	Budva	August 1955	-	-	-	-
	Budva	31 January 1998	185	80	-	juvenile
	Bar	5 August 2011	220	150	male	juvenile
Slovenia	Izola	22 October 1963	490–550	1100	-	adult
North Adriatic Sea		Before 1873	460			adult
Central Adriatic Sea		1969	-	-	-	-
Eastern Adriatic Sea		1901	500	-	-	adult
Eastern Adriatic Sea		May 1947	300	300	-	subadult
Adriatic Sea		Before 1919	438		female	subadult
Adriatic Sea		Before 1969	600			adult
